# *N*-(4-Meth­oxy­phen­yl)-4-methyl­benzene­sulfonamide

**DOI:** 10.1107/S1600536810052633

**Published:** 2010-12-18

**Authors:** Mehmet Akkurt, Irfana Mariam, Ifrah Naseer, Islam Ullah Khan, Shahzad Sharif

**Affiliations:** a Department of Physics, Faculty of Sciences, Erciyes University, 38039 Kayseri, Turkey; b Department of Chemistry, Government College University, Lahore 54000, Pakistan

## Abstract

In the title compound, C_14_H_15_NO_3_S, the dihedral angle between the aromatic rings is 59.39 (14)° and the C—S—N—C torsion angle is −71.4 (2)°. In the crystal, a supra­molecular chain running along the *b* axis with a *C*(4) graph set is formed *via* N—H⋯O hydrogen bonds.

## Related literature

For the biological activity of sulfonamides, see: Korolkovas (1988 ▶); Mandell & Sande (1992 ▶). For some structural studies of sulfonamides, see: Khan, Akkurt *et al.* (2010 ▶); Khan, Sharif *et al.* (2010 ▶); Sharif *et al.* (2010 ▶). For graph-set notation, see: Bernstein *et al.* (1995 ▶).
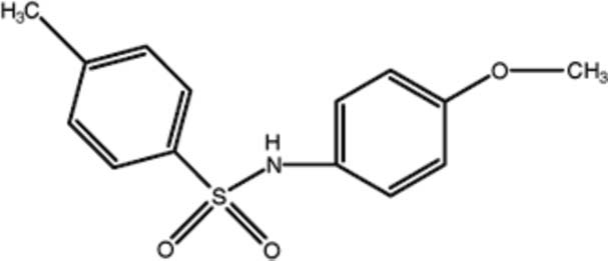


## Experimental

### 

#### Crystal data

C_14_H_15_NO_3_S
*M* *_r_* = 277.34
Monoclinic, *P*2_1_
*a* = 9.1777 (4) Å
*b* = 5.2179 (2) Å
*c* = 15.1621 (7) Å
β = 103.518 (2)°
*V* = 705.97 (5) Å^3^
*Z* = 2
Mo *K*α radiation
μ = 0.23 mm^−1^
*T* = 296 K
0.31 × 0.10 × 0.08 mm

#### Data collection

Bruker APEXII CCD diffractometer
6858 measured reflections
2995 independent reflections
2175 reflections with *I* > 2σ(*I*)
*R*_int_ = 0.028

#### Refinement

*R*[*F* ^2^ > 2σ(*F* ^2^)] = 0.043
*wR*(*F* ^2^) = 0.106
*S* = 1.03
2995 reflections
174 parameters
1 restraint
H-atom parameters constrained
Δρ_max_ = 0.22 e Å^−3^
Δρ_min_ = −0.19 e Å^−3^
Absolute structure: Flack (1983 ▶), 1078 Freidel pairs
Flack parameter: 0.01 (8)

### 

Data collection: *APEX2* (Bruker, 2007 ▶); cell refinement: *SAINT* (Bruker, 2007 ▶); data reduction: *SAINT*; program(s) used to solve structure: *SHELXS97* (Sheldrick, 2008 ▶); program(s) used to refine structure: *SHELXL97* (Sheldrick, 2008 ▶); molecular graphics: *ORTEP-3 for Windows* (Farrugia, 1997 ▶); software used to prepare material for publication: *WinGX* (Farrugia, 1999 ▶) and *PLATON* (Spek, 2009 ▶).

## Supplementary Material

Crystal structure: contains datablocks global, I. DOI: 10.1107/S1600536810052633/is2644sup1.cif

Structure factors: contains datablocks I. DOI: 10.1107/S1600536810052633/is2644Isup2.hkl

Additional supplementary materials:  crystallographic information; 3D view; checkCIF report

## Figures and Tables

**Table 1:** Hydrogen-bond geometry (Å, °)

*D*—H⋯*A*	*D*—H	H⋯*A*	*D*⋯*A*	*D*—H⋯*A*
N1—H1*A*⋯O2^i^	0.86	2.37	2.975 (3)	128

Symmetry code: (i) 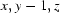.

**Fig. 1.**
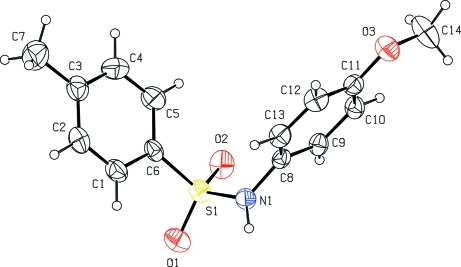
A view of the molecular structure of the title compound, showing the atomic numbering scheme. Displacement ellipsoids are drawn at the 30% probability level.

**Fig. 2.**
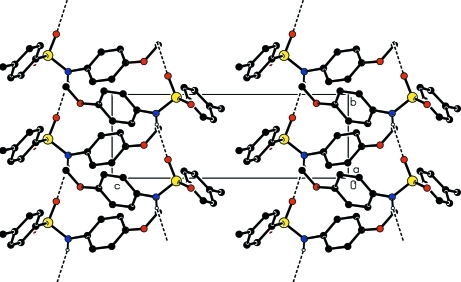
A view of intermolecular N—H···O hydrogen bonds connecting the molecules into an infinite one-dimensional chain extending along the b axis of the unit cell. For the sake of clarity, the H atoms not involved in the motif have been omitted.

## supplementary crystallographic information

### Comment

The crystal structure of the title compound, (I), was determined in continuation of structural studies of sulfonamides (Sharif *et al.*, 2010; Khan, Akkurt *et al.*, 2010; Khan, Sharif *et al.*, 2010), of interest owing to their enormous potential as biologically active molecules (Korolkovas, 1988; Mandell & Sande, 1992).

In the title compound (I), (Fig. 1), the dihedral angle between the two aromatic rings (C1–C6) and (C8–C13) is 59.39 (14)°. The molecule is twisted at the S atom with the C6—S1—N1—C8 torsion angle of -71.4 (2)°. The packing of molecules linked by of N—H···O hydrogen bonds (Table 1) form supramolecular chains [C(4) graph set; Bernstein *et al.*, 1995] aligned along the *b* axis (Fig. 2).

### Experimental

To *para* anisidine (123 mg, 1 mmol) in distilled water (10 ml) was added *para* toluene sulfonyl chloride (190 mg, 1 mmol) with stirring at room temperature while maintaining the pH of the reaction mixture at 8 using 3% sodium carbonate. The progress of the reaction was monitored by TLC. The product was dissolved in methanol and recrystallized by slow evaporation of the solvent, to generate colourless needles (I) in 79% yield.

### Refinement

All H atoms were positioned geometrically, with N—H = 0.86 Å, and C—H = 0.93 (aromatic H) and 0.96 Å (methyl H) and were refined using a riding model, with *U*_iso_(H) = 1.2 (1.5 for methyl groups)×*U*_eq_(C, N). Three low-angle reflections, (100), (-101) and (001), whose intensities were strongly affected by the beamstop, were omitted in the refinement process.

### Crystal data

**Table:**

C_14_H_15_NO_3_S	*F*(000) = 292
*M**_r_* = 277.34	*D*_x_ = 1.305 Mg m^−^^3^
Monoclinic, *P*2_1_	Mo *K*α radiation, λ = 0.71073 Å
Hall symbol: P 2yb	Cell parameters from 2249 reflections
*a* = 9.1777 (4) Å	θ = 2.4–24.3°
*b* = 5.2179 (2) Å	µ = 0.23 mm^−^^1^
*c* = 15.1621 (7) Å	*T* = 296 K
β = 103.518 (2)°	Needle, colourless
*V* = 705.97 (5) Å^3^	0.31 × 0.10 × 0.08 mm
*Z* = 2	

### Data collection

**Table:**

Bruker APEXII CCD diffractometer	2175 reflections with *I* > 2σ(*I*)
Radiation source: sealed tube	*R*_int_ = 0.028
graphite	θ_max_ = 28.3°, θ_min_ = 4.0°
φ and ω scans	*h* = −12→12
6858 measured reflections	*k* = −5→6
2995 independent reflections	*l* = −20→20

### Refinement

**Table:**

Refinement on *F*^2^	Secondary atom site location: difference Fourier map
Least-squares matrix: full	Hydrogen site location: inferred from neighbouring sites
*R*[*F*^2^ > 2σ(*F*^2^)] = 0.043	H-atom parameters constrained
*wR*(*F*^2^) = 0.106	*w* = 1/[σ^2^(*F*_o_^2^) + (0.0532*P*)^2^ + 0.0074*P*] where *P* = (*F*_o_^2^ + 2*F*_c_^2^)/3
*S* = 1.03	(Δ/σ)_max_ < 0.001
2995 reflections	Δρ_max_ = 0.22 e Å^−^^3^
174 parameters	Δρ_min_ = −0.19 e Å^−^^3^
1 restraint	Absolute structure: Flack (1983), 1078 Freidel pairs
Primary atom site location: structure-invariant direct methods	Flack parameter: 0.01 (8)

### Special details

**Table:**

Geometry. Bond distances, angles *etc*. have been calculated using the rounded fractional coordinates. All su's are estimated from the variances of the (full) variance-covariance matrix. The cell e.s.d.'s are taken into account in the estimation of distances, angles and torsion angles
Refinement. Refinement on *F*^2^ for ALL reflections except those flagged by the user for potential systematic errors. Weighted *R*-factors *wR* and all goodnesses of fit *S* are based on *F*^2^, conventional *R*-factors *R* are based on *F*, with *F* set to zero for negative *F*^2^. The observed criterion of *F*^2^ > σ(*F*^2^) is used only for calculating -*R*-factor-obs *etc*. and is not relevant to the choice of reflections for refinement. *R*-factors based on *F*^2^ are statistically about twice as large as those based on *F*, and *R*-factors based on ALL data will be even larger.

### Fractional atomic coordinates and isotropic or equivalent isotropic displacement parameters (Å^2^)

**Table:**

	*x*	*y*	*z*	*U*_iso_*/*U*_eq_	
S1	0.81437 (7)	0.96578 (12)	0.72795 (4)	0.0537 (2)	
O1	0.8984 (2)	0.8808 (4)	0.66620 (15)	0.0765 (8)	
O2	0.8331 (2)	1.2212 (3)	0.76342 (15)	0.0703 (7)	
O3	0.6677 (2)	0.8944 (4)	1.13469 (14)	0.0716 (8)	
N1	0.8557 (2)	0.7730 (4)	0.81409 (15)	0.0525 (7)	
C1	0.5796 (3)	0.7294 (6)	0.61403 (19)	0.0629 (10)	
C2	0.4331 (3)	0.6996 (6)	0.5708 (2)	0.0701 (11)	
C3	0.3249 (3)	0.8611 (6)	0.5875 (2)	0.0660 (11)	
C4	0.3684 (4)	1.0479 (7)	0.6502 (3)	0.0822 (16)	
C5	0.5157 (3)	1.0831 (6)	0.6953 (2)	0.0723 (11)	
C6	0.6241 (3)	0.9244 (4)	0.67623 (16)	0.0475 (8)	
C7	0.1637 (4)	0.8293 (9)	0.5376 (3)	0.1073 (18)	
C8	0.8041 (3)	0.8134 (4)	0.89527 (17)	0.0443 (8)	
C9	0.8648 (3)	1.0074 (5)	0.95370 (17)	0.0486 (8)	
C10	0.8214 (3)	1.0414 (4)	1.03440 (18)	0.0520 (9)	
C11	0.7174 (3)	0.8782 (5)	1.05675 (18)	0.0518 (9)	
C12	0.6576 (3)	0.6828 (5)	0.9981 (2)	0.0612 (10)	
C13	0.6996 (3)	0.6528 (5)	0.91731 (19)	0.0554 (9)	
C14	0.7243 (5)	1.0919 (8)	1.1956 (2)	0.0991 (16)	
H1	0.65090	0.61710	0.60160	0.0750*	
H1A	0.91080	0.64140	0.81100	0.0630*	
H2	0.40590	0.56740	0.52900	0.0840*	
H4	0.29590	1.15660	0.66330	0.0990*	
H5	0.54160	1.21270	0.73820	0.0870*	
H7A	0.14930	0.90180	0.47800	0.1610*	
H7B	0.10020	0.91560	0.57020	0.1610*	
H7C	0.13890	0.65040	0.53280	0.1610*	
H9	0.93550	1.11660	0.93890	0.0580*	
H10	0.86230	1.17380	1.07340	0.0620*	
H12	0.58850	0.57070	1.01320	0.0730*	
H13	0.65700	0.52320	0.87750	0.0660*	
H14A	0.70550	1.25390	1.16510	0.1490*	
H14B	0.83020	1.06920	1.21830	0.1490*	
H14C	0.67580	1.08790	1.24530	0.1490*	

### Atomic displacement parameters (Å^2^)

**Table:**

	*U*^11^	*U*^22^	*U*^33^	*U*^12^	*U*^13^	*U*^23^
S1	0.0675 (4)	0.0424 (3)	0.0518 (4)	−0.0033 (3)	0.0152 (3)	−0.0013 (3)
O1	0.0832 (13)	0.0850 (16)	0.0698 (14)	0.0004 (11)	0.0353 (12)	−0.0016 (11)
O2	0.0908 (14)	0.0416 (10)	0.0731 (13)	−0.0108 (9)	0.0080 (12)	0.0012 (10)
O3	0.0800 (13)	0.0754 (15)	0.0635 (13)	−0.0063 (10)	0.0249 (12)	−0.0036 (11)
N1	0.0632 (13)	0.0420 (12)	0.0494 (13)	0.0113 (9)	0.0075 (11)	−0.0029 (10)
C1	0.0763 (19)	0.0586 (16)	0.0544 (17)	0.0027 (15)	0.0166 (16)	−0.0142 (15)
C2	0.083 (2)	0.0731 (19)	0.0519 (18)	−0.0090 (17)	0.0109 (16)	−0.0158 (16)
C3	0.0721 (19)	0.076 (2)	0.0511 (17)	−0.0042 (16)	0.0169 (16)	0.0056 (16)
C4	0.074 (2)	0.083 (3)	0.092 (3)	0.0204 (16)	0.024 (2)	−0.010 (2)
C5	0.080 (2)	0.0585 (16)	0.076 (2)	0.0045 (16)	0.0133 (18)	−0.0241 (16)
C6	0.0703 (15)	0.0377 (14)	0.0355 (13)	0.0045 (11)	0.0147 (11)	0.0029 (10)
C7	0.071 (2)	0.158 (4)	0.090 (3)	−0.015 (2)	0.013 (2)	−0.010 (3)
C8	0.0457 (13)	0.0346 (12)	0.0471 (15)	0.0084 (11)	−0.0004 (12)	0.0002 (11)
C9	0.0503 (13)	0.0434 (16)	0.0479 (14)	−0.0063 (11)	0.0033 (12)	−0.0019 (12)
C10	0.0572 (15)	0.0453 (16)	0.0490 (16)	−0.0038 (11)	0.0032 (13)	−0.0063 (11)
C11	0.0491 (14)	0.0518 (16)	0.0515 (16)	0.0053 (11)	0.0060 (13)	0.0009 (12)
C12	0.0594 (15)	0.0503 (15)	0.074 (2)	−0.0097 (13)	0.0157 (15)	0.0007 (14)
C13	0.0566 (15)	0.0403 (14)	0.0644 (19)	−0.0071 (12)	0.0043 (14)	−0.0096 (13)
C14	0.144 (3)	0.098 (3)	0.066 (2)	−0.026 (3)	0.046 (2)	−0.019 (2)

### Geometric parameters (Å, °)

**Table:**

S1—O1	1.416 (2)	C10—C11	1.379 (4)
S1—O2	1.4324 (18)	C11—C12	1.380 (4)
S1—N1	1.622 (2)	C12—C13	1.376 (4)
S1—C6	1.752 (3)	C1—H1	0.9300
O3—C11	1.365 (3)	C2—H2	0.9300
O3—C14	1.400 (4)	C4—H4	0.9300
N1—C8	1.434 (3)	C5—H5	0.9300
N1—H1A	0.8600	C7—H7A	0.9600
C1—C6	1.383 (4)	C7—H7B	0.9600
C1—C2	1.360 (4)	C7—H7C	0.9600
C2—C3	1.370 (4)	C9—H9	0.9300
C3—C7	1.505 (5)	C10—H10	0.9300
C3—C4	1.355 (5)	C12—H12	0.9300
C4—C5	1.377 (5)	C13—H13	0.9300
C5—C6	1.376 (4)	C14—H14A	0.9600
C8—C13	1.372 (4)	C14—H14B	0.9600
C8—C9	1.375 (3)	C14—H14C	0.9600
C9—C10	1.384 (4)		
			
O2···C9	3.043 (3)	H1A···H10^iv^	2.3900
O2···N1^i^	2.975 (3)	H1A···H14B^iv^	2.5500
O1···H7B^ii^	2.6200	H2···H7C	2.5000
O1···H1	2.6400	H2···C1^ix^	2.8200
O2···H1A^i^	2.3700	H2···C2^ix^	3.0300
O2···H5	2.6100	H4···H7B	2.3700
O2···H9	2.6600	H5···O2	2.6100
N1···O2^iii^	2.975 (3)	H7B···O1^x^	2.6200
N1···H10^iv^	2.8000	H7B···H4	2.3700
C4···C14^v^	3.575 (6)	H7C···H2	2.5000
C9···O2	3.043 (3)	H9···O2	2.6600
C14···C4^vi^	3.575 (6)	H9···C9^viii^	2.9600
C1···H2^vii^	2.8200	H10···C14	2.5100
C2···H2^vii^	3.0300	H10···H14A	2.2600
C3···H14C^v^	2.9100	H10···H14B	2.3500
C4···H14C^v^	2.9600	H10···N1^viii^	2.8000
C8···H10^iv^	3.0700	H10···C8^viii^	3.0700
C9···H9^iv^	2.9600	H10···H1A^viii^	2.3900
C10···H14B	2.7700	H12···C11^v^	2.9400
C10···H1A^viii^	3.0200	H12···C12^v^	3.0100
C10···H14A	2.7000	H14A···C10	2.7000
C11···H12^vi^	2.9400	H14A···H10	2.2600
C12···H12^vi^	3.0100	H14B···C10	2.7700
C14···H10	2.5100	H14B···H10	2.3500
H1···O1	2.6400	H14B···H1A^viii^	2.5500
H1A···O2^iii^	2.3700	H14C···C3^vi^	2.9100
H1A···C10^iv^	3.0200	H14C···C4^vi^	2.9600
			
O1—S1—O2	120.23 (12)	C8—C13—C12	120.3 (2)
O1—S1—N1	106.10 (12)	C2—C1—H1	120.00
O1—S1—C6	107.78 (12)	C6—C1—H1	119.00
O2—S1—N1	106.85 (12)	C1—C2—H2	119.00
O2—S1—C6	107.59 (11)	C3—C2—H2	119.00
N1—S1—C6	107.76 (11)	C3—C4—H4	119.00
C11—O3—C14	118.0 (2)	C5—C4—H4	119.00
S1—N1—C8	122.56 (16)	C4—C5—H5	120.00
C8—N1—H1A	119.00	C6—C5—H5	120.00
S1—N1—H1A	119.00	C3—C7—H7A	109.00
C2—C1—C6	120.9 (3)	C3—C7—H7B	109.00
C1—C2—C3	121.2 (3)	C3—C7—H7C	109.00
C4—C3—C7	121.7 (3)	H7A—C7—H7B	109.00
C2—C3—C4	117.7 (3)	H7A—C7—H7C	110.00
C2—C3—C7	120.6 (3)	H7B—C7—H7C	109.00
C3—C4—C5	122.6 (3)	C8—C9—H9	120.00
C4—C5—C6	119.3 (3)	C10—C9—H9	120.00
S1—C6—C5	121.71 (19)	C9—C10—H10	120.00
C1—C6—C5	118.3 (3)	C11—C10—H10	120.00
S1—C6—C1	120.0 (2)	C11—C12—H12	120.00
C9—C8—C13	119.6 (2)	C13—C12—H12	120.00
N1—C8—C9	119.7 (2)	C8—C13—H13	120.00
N1—C8—C13	120.6 (2)	C12—C13—H13	120.00
C8—C9—C10	120.5 (2)	O3—C14—H14A	109.00
C9—C10—C11	119.8 (2)	O3—C14—H14B	110.00
O3—C11—C12	116.2 (2)	O3—C14—H14C	109.00
O3—C11—C10	124.4 (2)	H14A—C14—H14B	110.00
C10—C11—C12	119.5 (3)	H14A—C14—H14C	109.00
C11—C12—C13	120.4 (3)	H14B—C14—H14C	109.00
			
O1—S1—N1—C8	173.41 (19)	C1—C2—C3—C7	178.6 (3)
O2—S1—N1—C8	44.0 (2)	C7—C3—C4—C5	−178.7 (3)
C6—S1—N1—C8	−71.4 (2)	C2—C3—C4—C5	1.4 (5)
N1—S1—C6—C5	96.3 (2)	C3—C4—C5—C6	0.3 (5)
O2—S1—C6—C1	160.9 (2)	C4—C5—C6—S1	177.6 (3)
N1—S1—C6—C1	−84.2 (2)	C4—C5—C6—C1	−2.0 (4)
O1—S1—C6—C1	29.9 (2)	N1—C8—C13—C12	175.9 (2)
O2—S1—C6—C5	−18.6 (3)	C9—C8—C13—C12	−1.1 (4)
O1—S1—C6—C5	−149.7 (2)	N1—C8—C9—C10	−176.9 (2)
C14—O3—C11—C10	0.9 (4)	C13—C8—C9—C10	0.1 (4)
C14—O3—C11—C12	−179.3 (3)	C8—C9—C10—C11	0.5 (4)
S1—N1—C8—C9	−72.4 (3)	C9—C10—C11—C12	−0.1 (4)
S1—N1—C8—C13	110.6 (2)	C9—C10—C11—O3	179.7 (2)
C2—C1—C6—C5	1.9 (4)	O3—C11—C12—C13	179.3 (2)
C6—C1—C2—C3	−0.1 (5)	C10—C11—C12—C13	−0.9 (4)
C2—C1—C6—S1	−177.7 (2)	C11—C12—C13—C8	1.5 (4)
C1—C2—C3—C4	−1.5 (5)		

Symmetry codes: (i) *x*, *y*+1, *z*; (ii) *x*+1, *y*, *z*; (iii) *x*, *y*−1, *z*; (iv) −*x*+2, *y*−1/2, −*z*+2; (v) −*x*+1, *y*−1/2, −*z*+2; (vi) −*x*+1, *y*+1/2, −*z*+2; (vii) −*x*+1, *y*+1/2, −*z*+1; (viii) −*x*+2, *y*+1/2, −*z*+2; (ix) −*x*+1, *y*−1/2, −*z*+1; (x) *x*−1, *y*, *z*.

### Hydrogen-bond geometry (Å, °)

**Table:**

*D*—H···*A*	*D*—H	H···*A*	*D*···*A*	*D*—H···*A*
N1—H1A···O2^iii^	0.86	2.37	2.975 (3)	128

Symmetry codes: (iii) *x*, *y*−1, *z*.
